# Assessment of segmentation and targeted counseling on family planning quality of care and client satisfaction: a facility-based survey of clients in Niger

**DOI:** 10.1186/s12913-021-07066-z

**Published:** 2021-10-11

**Authors:** Ilene S. Speizer, Hachimou Amani, Jennifer Winston, Souleymane Amadou Garba, Amelia Maytan-Joneydi, Illiassou Chaibou Halidou, Lisa M. Calhoun, Abdoul Moumouni Nouhou

**Affiliations:** 1 Department of Maternal and Child Health, Gillings School of Global Public Health, University of North Carolina at Chapel Hill, Chapel Hill, NC USA; 2 Carolina Population Center, University of North Carolina at Chapel Hill, Chapel Hill, NC USA; 3 Grade Africa, Niamey, Niger

**Keywords:** Family Planning, Quality of care, Satisfaction, Segmentation, Niger

## Abstract

**Background:**

Niger demonstrates high fertility and low contraceptive use that are typical in much of the West and Central African region. The government of Niger has committed to increasing modern contraceptive use as part of its health strategy. Designing and testing strategies to improve quality of care and satisfaction of family planning clients is important for addressing low contraceptive use in contexts like Niger.

**Methods:**

This study uses recently collected client exit interview data from 2720 clients surveyed in the Dosso region of Niger to examine whether implementation of segmentation-based counseling leads to improved quality of services and client satisfaction. We compare three scenarios: a) facilities where segmentation counseling was implemented since 2017; b) facilities where segmentation counseling began in late 2019; and c) facilities without segmentation counseling. Bivariate and multivariate analyses are undertaken to determine if there are differences in quality of services and client satisfaction between the facility groups and between clients that were segmented and those who were not segmented in the first two scenarios.

**Results:**

Results demonstrate that clients in facilities with segmentation generally received better quality services than clients in facilities without segmentation. Clients in facilities implementing segmentation longer reported higher quality services than the recent segmentation facilities. Clients who were segmented compared to those who were not segmented also reported better quality services. New clients reported higher quality services than returning clients and among new clients, those who were segmented also reported higher quality services. No differences were found in client satisfaction between facility scenarios or between segmented and non-segmented clients.

**Conclusions:**

These findings demonstrate that segmentation or another targeted counseling strategy could be useful to the government of Niger to improve the quality of services offered. As part of the scale up process, the government needs to consider strategies that ensure that all new clients are segmented and design an approach that is sustainable and does not risk failing should there be stock-out of segmentation sheets or loss of counseling cards. This type of targeted counseling could improve the quality of services offered and ideally lead to increased contraceptive use in Niger.

## Background

Contraceptive use remains low in many parts of West and Central Africa. This is the case in the site of this study, Niger, a landlocked country in the Sahel that is more than 98% Muslim and had a 2021 estimated population of 25.1 million [1]. With an average of 7.6 children per woman in 2012 [2], Niger had the highest total fertility rate (TFR) in the world. In 2017, the percentage of Nigerien women of reproductive age using a modern method of contraception was 15.2% overall and 18.1% among married women [3]. Furthermore, with 50% of the population under the age of 15 [1], Niger is facing the challenge of rapid population growth and significant population momentum. Approximately one in four adolescents ages 15–19 are married by the age of 15, and three in four women ages 20–24 are married before they turn 18 [2]. Half of women ages 25–49 had their first birth before age 19 and 48% of adolescents ages 15–19 had already had a live birth or were currently pregnant at the time of the 2012 Demographic and Health Survey [2]. The young age at first marriage and childbearing and the fact that 12% of women have an unmet need for family planning for spacing purposes [2] indicate the need for strong family planning programs in Niger.

Helping women to access quality family planning (FP) services is an important part of meeting Sustainable Development Goal 3 and meeting Family Planning 2020 (now Family Planning 2030) commitments to increase modern FP use. The government of Niger committed at the 2012 London Summit on Family Planning to achieving a modern contraceptive prevalence of 50% by 2020. At the follow-up summit in 2017, the government of Niger also committed to expanding access to implants and injectables through community-based distribution and to increasing attention for adolescent and youth sexual and reproductive health needs [4]. The 2017 estimate of 18% contraceptive prevalence among married women [3] falls well short of the target set in 2012.

Numerous reasons for the low use of contraception in Niger and other parts of West and Central Africa have been identified including continued desire for large families [5], lack of access to FP services, fear of side effects or health effects, and opposition to FP use [6, 7]. In addition, provider bias, that is providers choosing not to offer FP methods (or specific types of methods) to women based on age, parity, and marital status, affects use in many countries [8].

To address these challenges, many initiatives have focused on improving the quality of FP services as a human right and a fundamental principle of FP service provision [9]. In 1990, Bruce [10] defined quality of FP programming in terms of six key elements: choice of methods, information provided to clients, service provider competence, interpersonal relations, continuity and follow-up mechanisms, and appropriate constellation of services. Although programs that affect these elements of quality of services may not directly increase contraceptive use levels or affect fertility outcomes [11–13], they can be important for creating demand for FP services or addressing the other reasons for non-use of contraception. For example, improved quality can lead to increased use by: a) addressing rumors and myths about FP; b) improving client satisfaction resulting in positive perceptions of services at the community level; c) ensuring that facilities are well equipped to provide a range of FP methods to meet the varying needs of women in their communities; d) reducing provider bias; and e) providing FP services in an integrated manner to support use among new mothers who have been identified to have a need for FP [14, 15].

A key approach to address quality of FP services has been to train providers to improve provider counseling skills. Provider training on a broad range of contraceptive methods, interpersonal skills, provider bias, and method-specific side effects can address many of the Bruce [10] elements of quality of care including a) choice of methods, b) information provided to clients, c) interpersonal relations, and d) follow-up and continuity mechanisms. Evidence on the effectiveness of provider training on quality of care and client satisfaction is limited.

One approach to improve provider-client interactions developed by the Population Council in the late 1990s is the Balanced Counseling Strategy (BCS). This approach has three stages: the pre-choice stage, the method choice stage, and the post-choice stage. It uses an algorithm to guide the provider through the counselling process and provides resources to help the client make an informed FP decision [16]. This approach was first implemented and evaluated in Peru and Guatemala and was used to design the Balanced Counseling Strategy Plus (BCS+) Toolkit. Evaluation results of the BCS in Peru demonstrated higher client knowledge among clients who consulted providers that used the BCS job aids than when they consulted providers who did not use the job aids [16]. In Guatemala, using mystery clients, a study found better quality of care outcomes related to interpersonal relations, choice of methods, provision of information on contraindications of methods, and detailed information provided on the selected method in clinics that adopted the BCS approach than those that did not; however, distinctions were also found in the experimental group based on the intensity of the trainings offered [16, 17]. Notably, at one-year follow-up, results from Peru showed greater knowledge among clients who were exposed to the BCS; however, the authors could not separate out the effect of the BCS and the method pamphlets shared with clients that may have served as a memory aid [18].

Further tests of employing balanced counseling (BC) approaches have been undertaken more recently. The overall goal of these BC approaches is to tailor counseling to the individual client’s specific reproductive health needs and circumstances so that they can choose a method that best meets their needs [16, 19]. The BC approach has been tested in various country settings (Nepal, Tanzania, and Indonesia) using different implementation approaches. For example, in Nepal, an algorithm was used to assess women’s reproductive goals and their contraceptive needs and based on the output of this algorithm, contraceptive details were provided by the provider based on BC cards [19]. In Tanzania, the BC approach was part of a mobile job aid for counseling by community health workers (CHW); the mobile job aid included the BCS+ strategy among other tools and checklists [20, 21]. Further, in Indonesia, BCS was used with postpartum clients [22]. Across these implementation approaches, there were generally positive findings including greater adoption of long-acting reversible contraceptives (LARC) over time in Nepal where the BC approach was implemented [19]; high acceptability of the job aids by the CHW and clients were knowledgeable about more methods after program implementation in Tanzania [21]; and increased postpartum FP counseling where BCS was used in Indonesia [22]. Generally, these studies examined trends over time, but none included a comparison group. In Niger, the site of this study, L’Initiative OASIS improved maternal and neonatal health services by undertaking group antenatal and postnatal care by identifying homogenous groups of women for the sessions; this strategy is similar to BCS because it focuses on targeting the approach to homogenous groups of women. The Niger team found that 51.9% of women participating accepted a postpartum FP method; this compares to 22% of women using at six months postpartum from national estimates [23]. No studies were found in Niger or elsewhere that tested the relationship between targeted client counseling and client satisfaction and none of the recent studies found included intervention and comparison sites; these are contributions of this study.

Another approach used to provide services in a more tailored manner to clients is segmentation. Segmentation may be done based on demographic, economic, or geographic characteristics of clients. While market segmentation is a common approach, segmentation of the FP market has been less commonly employed. FP market segmentation has been undertaken for USAID to better understand non-user or potential user groups in Azerbaijan [24], Ghana [25], and Ethiopia [26], among other sites. In Niger, the site of this study, Camber Collective has undertaken segmentation studies of women and men.^1^ Based on survey information on previous health seeking behaviors, social norms, contraceptive attributes, and attitudes and beliefs, Camber Collective developed a rapid segmentation tool to segment clients and provide them with targeted counseling (see intervention description below).

### Objective of this study

Camber Collective, under Pathfinder International’s IMPACT project, developed a segmentation tool and counselling cards that have been implemented by Pathfinder International in facilities offering FP services. The objective of this study is to assess the quality of services and the level of client satisfaction with FP services received as reported by clients in sites where segmentation tools were implemented. The information from this assessment is crucial to the Government of Niger to inform scale-up of the tool and counseling cards throughout Niger. The results of this study are also useful for determining the utility of this tool for other programs outside of the target region and country. This assessment was carried out by the Full Access, Full Choice project which is implemented in Niger through a partnership between L’Initiative OASIS Niger (now GRADE Africa) and the University of North Carolina at Chapel Hill Carolina Population Center (CPC). These partners worked in collaboration with Pathfinder International (the implementer) and the Government of Niger (Direction de la Planification Familiale).

### Segmentation intervention in Niger

The main intervention being examined in this study is a novel client segmentation counseling tool developed by Camber Collective and implemented by Pathfinder International. Particularly in resource-constrained settings, segmentation, or the division of a population into subgroups based on shared defining characteristics, can aid policymakers and programs to focus finite resources on the most receptive segments within a population. In 2014, Camber Collective undertook a nationally representative survey of over 2000 women aged 15–49 in Niger. The data from this survey were segmented by five key factors: contraceptive use behaviors, proactivity, social norms, contraceptive attributes, and attitudes and beliefs. The analysis yielded five target segments: Healthy Proactives, Traditional Autonomists, Sheltered Skeptics, Modern Elites, and Conservative Passives [27]. In their final report, Camber proposed that tailored programming to priority segments and improved quality of health worker counseling were two ways to increase contraceptive use in Niger [27].

As a follow-up, Camber Collective developed a segmentation counseling tool and different counseling cards specific to each of the five segments. The client segmentation tool guides providers to ask 12 questions to determine which of the five segments a client falls into. Providers then use counseling cards to tailor FP counseling to clients based on their identified segment. The counseling cards outline potential attitudes towards and concerns with FP, points to be discussed with clients, messages that are likely to resonate with clients, and types of methods clients may be more likely to accept. This approach is particularly useful in Niger where there are still high fertility desires and contraceptive use is not a normative behavior; certain segments are likely the innovators (e.g., Modern Elites and Heathy Proactives) whereas others still want many children and are likely to be resistant to contraceptive use (Traditional Autonomists and Sheltered Skeptics).

Pathfinder International introduced segmentation into Dosso region of Niger in two phases. First, Pathfinder International incorporated segmentation into facilities participating in their Reaching Married Adolescents (RMA) project, which seeks to address demand-side factors for FP among married adolescents. Pathfinder trained one provider in each study area health facility to use the counseling tool and counseling cards through a five-day training. Second, in a sub-set of facilities without RMA, Pathfinder International incorporated the segmentation tool and counseling cards and provided a training for providers.

## Methods

### Study goal and sampling design

There are two primary aims of this study. The first aim is to assess whether services are of higher quality in facilities using the segmentation tool and counseling cards. The second aim is to assess whether clients in facilities that are using the client segmentation tool and counseling cards are more satisfied than clients in facilities that are not using these tools. In addition, focusing on clients in the intervention arms, we examine if clients who are segmented receive higher quality services or have higher satisfaction than clients who are not segmented.

The study takes place in integrated health centers (Centre de Santé Intégré - CSI) in six districts of Dosso region in Niger. CSIs are free-standing public sector health centers that serve much of the population with all essential primary healthcare services including antenatal care, delivery services, postnatal care, well-baby care, and family planning services. CSIs generally serve a population of around 25,000 people. There are two types of CSIs, type 1 and type 2; typically type 1 CSIs are smaller and serve a smaller client load than type 2 CSIs. CSIs are found in both urban and rural areas; however, the CSIs included in the Dosso region for this study are mostly in rural areas serving the population that is predominately rural.

The overall study of the client segmentation counseling tool includes mixed methods that include a facility audit, in-depth interviews (IDIs) with providers, and client exit interviews. The focus of this paper is the quantitative results from the client exit interviews.

The study uses a quasi-experimental design with three different implementation scenarios (or arms) in 45 CSIs in Boboye, Doutchi, Dosso, Falmey, Loga, and Tibiri health districts of Dosso region. At project launch, there were 15 CSIs in Arm 1 that were undertaking segmentation since 2017 as part of the RMA project, therefore it was not possible to randomize CSIs by arm. The CSIs in Arms 2 and 3 were assigned after identification of the 40 eligible non-RMA CSIs in the target health districts. Since in Arm 1 there were eight type 1 CSIs and seven type 2 CSIs, our sampling objective was to include a similar distribution of type 1 and type 2 CSIs in Arms 2 and 3 to reduce variability by size of facility. The three arms provide differing perspectives on implementation of the segmentation tool and counseling cards to inform future government roll-out of the tool. The three study arms are as follows:

#### Arm 1 (RMA and segmentation)

15 CSI sites where RMA and the segmentation tool and counseling cards have been implemented (these sites have had segmentation since 2017)

#### Arm 2 (segmentation only)

15 CSI sites where only the segmentation tool and counseling cards were implemented (without RMA). Training and implementation of the segmentation approach began in October 2019

#### Arm 3 (control)

15 CSI sites where there is no RMA nor segmentation

In the Arm 3 scenario where there was no training, services continued to be offered in the standard approach. Typically, counseling sessions are done in groups for all clients. The FP providers present the methods and explain their advantages and disadvantages and then clients take turns entering the FP room to make their choices based on the explanations provided by the provider; generally, clients are served without in-depth discussion.

The facilities included across the three arms are similar to one another. In each Arm, there are 8 type 1 CSIs (lower volume) and 7 type 2 CSIs. On average, there are 3.4 midwives per facility with slightly more in Arm 1 (3.67) and slightly fewer in Arm 2 (3.13) with Arm 3 in the middle (3.40). On average the facilities had 434 clients that received FP counseling or services in the last three months. This number was highest in Arm 2 (474) and lowest in Arm 1 (368). On average, 80% of the facilities have an information, education, and communication program. This is lowest in Arm 1 (73%) compared to Arm 2 (80%) and Arm 3 (87%). All but one facility in the sample is open seven days a week and all facilities offer antenatal care and FP services; 43 of the 45 facilities offer post-abortion care. All facilities offer the main contraceptive methods used in Niger including the pill, condom, injectable, and implant; all but one facility offers the IUD. Thus, generally, the public sector CSIs in the study sample are similar across the study arms in the services they provide, their size, and the methods offered.

### Data and measures

In each of the 45 study CSIs, one or two interviewers from the Dosso region were assigned to interview all FP clients during a 2-month period. Project supervisors who were all from Niger and spoke the local languages of the Dosso region were trained by L’Initiative OASIS (now GRADE Africa) and CPC in January 2020 in Niamey, and interviewers from the Dosso region with experience undertaking this type of data collection were subsequently trained by project supervisors and L’Initiative OASIS in Dosso in early February 2020 to implement the tablet-based survey. Data collection took place during 53 days in February and March 2020. Interviewers were withdrawn from study sites one week earlier than planned due to the COVID-19 pandemic. Because of this early departure, some interviewers were unable to complete verification of segmentation forms for some clients as discussed below.

All FP clients were approached by the project interviewers and read the consent form in their preferred local language (Hausa or Zarma) and asked for signed informed consent for participation in the study following their visit to the CSI. During the study period, only five women refused to be interviewed. In total, client exit interview surveys were collected from 2720 women: 679 from Arm 1, 898 from Arm 2, and 1143 from Arm 3 (see Table 1 for characteristics of samples). The exit interviews were implemented in Hausa or Zarma and took about 35 min to complete. Participating clients were thanked for their time but not given any compensation for participation. Approval for the study protocol, consent procedures, and consent forms was provided by the Niger Comité National d’Ethique pour la Recherche en Santé (CNERS) and by the Institutional Review Board at the University of North Carolina in Chapel Hill.

**Table 1:** Characteristics of the Segmentation Study sample by study arm, Dosso region, Niger, 2020

	Arm 1	Arm 2	Arm 3	Total
Characteristic	***n*** = 679^**a**^	***n*** = 898^**a**^	***n*** = 1143^**a**^	***n*** = 2720^**a**^
Use of FP at time of visit				
New user (non-user)	41.83	54.79	44.71	47.32
Continuing user	58.17	45.21	55.29	52.68
Age of the woman				
15–19	13.25	12.03	12.95	12.72
20–24	27.25	28.06	28.70	28.13
25–29	26.51	26.50	23.45	25.22
30–34	17.08	17.71	17.24	17.35
35–39	10.75	10.91	12.25	11.43
40+ years	5.15	4.79	5.42	5.15
Marital status				
Unmarried (divorced, widowed, never married, refusal)	0.24	2.45	1.22	1.50
Married or living with partner	99.26	97.55	98.78	97.50
Level of education*				
None	49.85	38.31	53.28	47.48
Quranic/literate	14.01	31.63	18.81	21.85
Primary	16.08	16.04	15.57	15.85
Secondary or higher	20.06	14.03	12.34	14.82
Parity				
0–1	22.42	19.09	20.18	20.38
2	20.33	21.26	22.95	21.73
3	17.34	17.71	17.14	17.38
4	14.35	15.09	14.64	14.71
5	12.26	10.97	10.80	11.22
6+	13.30	15.89	14.29	14.56
Segmented*				
No	29.01	49.44	NA	
Yes	49.19	44.65	NA	
Missing	21.80	5.90	NA	
Received a method during visit				
No	6.25	11.11	6.75	8.08
Yes	93.75	88.89	93.25	91.92
Method received (among those who adopted)				
Implant	12.03	17.55	10.48	13.15
IUD	0.16	0.63	0.00	0.24
Injectable	56.26	53.54	62.95	58.24
Pill	31.54	28.28	26.38	28.29
Other	0.00	0.00	0.19	0.08

^a^Some n’s smaller due to a small amount of missing information

*p ≤ 0.05 for chi-square test between groups

The key outcome variables for this study are based on the quality of services received and client satisfaction. The measures used to create these outcomes and the distributions are presented in Table 2. For the quality of services, we include four variables: exposed to different FP methods using a demonstration kit, poster, and/or pictures (yes vs. no); given information on the range of methods (yes vs. no); asked about preferred method (yes vs. no); and information received about the chosen method during her interaction with the provider. This last variable was only measured for women who received a method; all other women were coded zero. Women who received a method were asked if they received information on: a) how to use the method, b) side effects of the method, c) what to do if she has problems with the method, and d) the possibility to change methods. Those clients who were told about all four of these factors are coded one and all others are coded zero. If a client reported that she did not know if she received any of the services, she was coded as “no.” Note that these quality questions were selected to capture two of the key Bruce [10] quality elements including choice of methods and information provided to clients. The other Bruce elements of provider competence, continuity and follow-up mechanisms, and constellation of services are not well captured through the client exit interviews. To permit examination across the four quality measures, we also created a quality score that was the average value of the four variables (ranges from 0 to 1); because some of the quality measures had missing data, we created the quality score based on the available data for each measure (i.e., if only three measures were non-missing, the average is based on those three variables). Note that generally the data for the quality measures were complete (i.e., 2% or less missing data), however, in Arm 1, there was slightly more missing data, particularly on the preference measure (3.2% missing) and the interaction with provider measure (4.1% missing) (contact first author for a table with level of missingness).

**Table 2:** Quality and satisfaction with services as reported by the clients by study arm, Dosso region, Niger, 2020

	Arm 1	Arm 2	Arm 3
	***n*** = 679^**a**^	***n*** = 898^**a**^	***n*** = 1143^**a**^
**Quality of counseling**			
Was given information about the different methods of FP (% yes)*	71.13	55.03	45.96
Was asked about her preference for a method of FP (% yes)*	87.06	73.32	74.76
The provider showed you the demonstration kit with the methods, the pictures of the methods, a poster with the methods or examples of the methods during your discussion (% yes)	40.48	37.18	20.14
Interaction with provider - About the method you received, provider told you: a) how to use the method; b) about side effects of the method; c) what to do if you have problems with the method; and d) that you could change methods) - (% yes to all four)*	46.39	36.27	20.23
Average quality score based on four items above (0–1)	0.60	0.50	0.40
**Level of satisfaction**			
Would you say that the time you spent waiting for your appointment was:			
No waiting time	33.43	34.23	22.05
Reasonable	47.04	52.80	54.42
Too long	19.53	12.98	23.53
During your visit, how were you treated by the provider? (% “very well”)	32.25	52.46	32.98
Are you very satisfied, satisfied, somewhat satisfied or not satisfied at all with your FP visit to the facility today? (% “very satisfied”)	25.67	44.68	27.56
Average satisfaction score based on three items (no waiting time/reasonable coded 1 vs. too long coded zero) (0–1)	0.46	0.61	0.46

^a^Some n’s smaller due to a small amount of missing information

*p ≤ 0.05 for chi-square test between groups

The other key outcome in this study is client satisfaction with services received. Selected satisfaction measures include client wait time, how the client perceived she was treated by the provider, and her overall satisfaction with the visit. Wait time was coded as one if the client reported no waiting time or a reasonable waiting time and coded as zero if she reported that the waiting time was too long. All women were asked how well they were treated by the family planning agent. They were also asked how satisfied they were with their visit to the facility on the day of interview. For these variables, a response of very well treated or very satisfied is coded as one and all other responses are coded as zero. As above, we created a satisfaction score based on the average of these three items (range 0–1); where there was missing data, the average was based on the available data. Note that for these satisfaction measures, less than 1% of the sample did not respond to the questions and the highest missingness was for Arm 1 for satisfaction with the visit at 0.74% missing a response (i.e., 5 clients).

To consider the role of segmentation in Arms 1 and 2, we also captured if the woman was segmented. The segmentation variable has three categories: yes, no, missing information. Following the client exit interview in Arms 1 and 2, interviewers checked each client’s folder to verify whether there was a segmentation sheet to determine whether the client had ever been segmented. Missing information on segmentation was a consequence of either the interviewer not being able to find the folder or the interviewer not having a chance to check the folder before data collection closed.

All analyses control for characteristics of the clients at the time of the visit. These include whether she was using a FP method at the time of the visit (i.e., continuing user compared to new users), the age of the woman, level of education, and parity. Very few women had no children, so women with 0 children and 1 child are grouped together. More than 99% of the clients were married or in union and thus marital status is not included in the models. In models of new users, we also include whether the woman had ever used family planning in the past (compared to never used). Distributions of these variables are presented in Table 1 by study arm.

### Analysis approach

Descriptive comparisons of the outcomes are first presented by study arm to assess whether clients visiting facilities with segmentation (Arms 1 and 2) receive higher quality services and are more satisfied than those visiting facilities without segmentation (Arm 3). Next, we examine whether in Arms 1 and 2, clients who were segmented report higher quality services and more satisfaction than clients who were not segmented. Multivariable analyses are performed to examine whether, controlling for if the client was a new/returning client, her age, education, and prior birth experience, study arm is associated with greater quality or satisfaction or if being segmented is associated with the outcomes of interest. We use logistic regression and present odds ratios and 95% confidence intervals for all of the outcomes that are coded as 0/1 variables. For the quality score and the satisfaction score, we use linear regression methods and present coefficients and standard errors. All multivariable analyses adjust the standard errors to control for the study design that is clustered at the CSI level.

## Results

Table 1 presents the descriptive characteristics of the study sample. Arm 3 had the most clients in the study period followed by Arm 2. Arm 1 had the fewest. This reflects the number of clients visiting the selected CSI for FP services and the size of the CSI. In Arms 1 and 3, more than half of the FP clients were continuing users; in Arm 2, 45.2% were continuing users. Notably, based on information provided by Pathfinder International, the segmentation tool was meant to be used only with new clients; however, in Arm 1, about 55% of returning clients had ever been segmented and in Arm 2, about 20% had been segmented (not shown). Because segmentation status was determined by looking at the participants’ charts, it should be noted that for clients from Arm 1, the segmentation recorded may have happened prior to the current visit (i.e., on the client’s first visit) since segmentation had been implemented since 2017. In Arm 2, since segmentation was newly implemented, providers may have been segmenting both new and returning clients in some of the CSIs on the client’s first visit since segmentation launch to have the relevant information for all clients.

The demographic characteristics of the samples by arm demonstrate no significant difference by age or parity by arm. Across the arms, most of the clients presenting for a FP visit are between the ages of 20–34 years (about 70%). In each arm, about 20% of the sample has no children or one child and about 20% have two children. Another 13–16% have six or more children. One significant difference observed across the three arms is by level of education where Arm 1 has the greatest percentage of clients who have secondary or higher education and Arm 3 has the most with no education. Across all three arms, about 16% have primary education.

Also presented in Table 1 is whether the client received a method on the day of the visit and what method was received for those clients receiving a method. The overwhelming majority of clients received a method; those who did not receive a method generally were visiting to discontinue a method to get pregnant or for another reasons (e.g., side effects). Among those who received a method, the main method received was the injectable (over 50% in all arms) followed by the pill. Fewer women received the implant; however, the percentage receiving the implant was highest in Arm 2 at 17.6%.

Table 2 presents the percentage of clients who report the different quality indicators and satisfaction measures by arm. Examining the quality indicators first, we see that clients from Arm 1 are significantly more likely to report that they were given information about different methods than clients in Arms 2 and 3. A significantly greater percentage of clients in Arm 1 report that they were asked about their preference for a method than in Arms 2 and 3. In addition, a significantly greater percentage of clients receiving a method in Arm 1 report that the provider gave full information on the method selected during their interaction. This includes receiving information on how to use the method, information about side effects, information on what to do if they have problems with the method, and information on how to change a method. There is no significant difference by arm in the provider showing clients the demonstration kit of the methods. It is worth noting that for three of the four quality indicators, while Arm 1 is the highest, Arm 2 is higher than Arm 3; the exception is for the indicator on being asked about a preference for a method where Arms 2 and 3 are similar. As expected, based on the discussion above, the quality score is highest in Arm 1 (0.60) and lowest in Arm 3 (0.40), with Arm 2 falling in between (0.50); the difference between Arm 1 and Arm 3 is significant.

In terms of wait time, Table 2 shows that the majority of the clients feel that they did not have to wait or the wait time was reasonable. In Arm 2, only 13% feel the wait time was too long compared to 19.5% in Arm 1 and 23.5% in Arm 3. More than 50% of clients in Arm 2 reported that the provider treated them very well whereas in Arms 1 and 3, only a third of clients reported that they were treated very well. Correspondingly, a greater percentage of clients in Arm 2 reported that they were very satisfied (44.7%) compared to Arm 3 (27.6%) and Arm 1 (25.7%). None of the satisfaction indicators are significantly different across arms. Corresponding to the descriptive satisfaction measures above, the satisfaction score is highest (0.61) in Arm 2, and Arms 1 and 3 are the same at 0.46; this difference is not significant.

Tables 3 and 4 present the bivariate analysis for each of the quality and satisfaction outcome variables and segmentation. These tables are limited to Arms 1 and 2, where segmentation took place. Table 3 presents these analyses among all clients in Arms 1 and 2 while Table 4 presents the same results for the sub-sample of new clients, since segmentation was intended to be targeted to this group. Because the team was not able to confirm segmentation status for a small number of clients, we include a “missing” category in these tables. Table 3 demonstrates that for three of the quality indicators (receiving information about different methods, being shown the demonstration kit, and receiving full information about the selected method), clients who were segmented report better quality of services than clients who were not segmented (or were missing the segmentation form); this is true in both Arm 1 and Arm 2. Further, only in Arm 2 do we find that segmented clients were significantly more likely to be asked about their method preference than non-segmented clients. As expected, segmented clients have significantly higher quality scores than non-segmented clients.

**Table 3:** Quality and satisfaction with services as reported by the clients by whether segmented or not, Arms 1 and 2, Dosso region, Niger, 2020

	Arm 1			Arm 2		
	Not segmented	Segmented	Missing	Not segmented	Segmented	Missing
**Quality of counseling**						
Was given information about the different methods of FP (% yes)	61.42	82.61	58.90*	32.27	83.38	29.41***
Was asked about her preference for a method of FP (% yes)	83.51	91.64	81.43	61.16	92.42	27.45***
The provider showed you the demonstration kit with the methods, the pictures of the methods, a poster with the methods or examples of the methods during your discussion (% yes)	21.43	56.00	31.51***	17.05	62.75	11.32***
Interaction with provider - About the method you received, provider told you: a) how to use the method; b) about side effects of the method; c) what to do if you have problems with the method; and d) that you could change methods) - (% yes to all four)	32.99	55.25	44.36**	22.30	53.65	20.75**
Average quality score (0–1)	0.49	0.70	0.52	0.33	0.73	0.22
**Level of satisfaction**						
Would you say that the time you spent waiting for your appointment was:						
No waiting time	29.95	30.21	45.27	32.05	35.91	39.62
Reasonable	48.73	50.45	37.16	55.00	50.12	54.72
Too long	21.32	19.34	17.57*	12.95	13.97	5.66
During your visit, how were you treated by the provider? (% “very well”)	29.95	33.53	32.43	52.50	48.38	83.02
Are you very satisfied, satisfied, somewhat satisfied or not satisfied at all with your FP visit to the facility today? (% “very satisfied”)	21.94	29.39	22.30	46.59	38.25	77.36+
Average satisfaction score (0–1)	0.43	0.48	0.46	0.62	0.58	0.85
Number of observations^a^	197	334	148	444	401	53

^a^Some n’s smaller due to a small amount of missing information. +*p* ≤ 0.10; *p ≤ 0.05; **p ≤ 0.01; ***p ≤ 0.001 for chi-square test between groups

**Table 4:** Quality and satisfaction with services as reported by new clients by whether segmented or not, Arms 1 and 2, Dosso region, Niger, 2020

	Arm 1			Arm 2		
	Not segmented	Segmented	Missing	Not segmented	Segmented	Missing
**Quality of counseling**						
Was given information about the different methods of FP (% yes)	78.87	91.18	66.67***	42.64	86.46	24.24***
Was asked about her preference for a method of FP (% yes)	90.00	97.08	72.22**	65.32	94.15	21.21***
The provider showed you the demonstration kit with the methods, the pictures of the methods, a poster with the methods or examples of the methods during your discussion (% yes)	22.54	64.91	44.44**	25.00	68.62	8.82***
Interaction with provider - About the method you received, provider told you: a) how to use the method; b) about side effects of the method; c) what to do if you have problems with the method; and d) that you could change methods) - (% yes to all four)	38.57	53.14	33.33	22.14	54.66	20.59*
Average quality score (0–1)	0.57	0.75	0.54	0.37	0.76	0.18
**Level of satisfaction**						
Would you say that the time you spent waiting for your appointment was:						
No waiting time	39.44	30.29	50.00	25.76	33.44	26.47
Reasonable	47.89	45.14	30.56	58.33	50.31	64.71
Too long	12.68	24.57	19.44	15.91	16.26	8.82
During your visit, how were you treated by the provider? (% “very well”)	42.25	30.29	36.11	57.58	48.47	94.12*
Are you very satisfied, satisfied, somewhat satisfied or not satisfied at all with your FP visit to the facility today? (% “very satisfied”)	28.17	28.74	22.22	48.48	38.46	88.24**
Average satisfaction score (0–1)	0.53	0.45	0.46	0.63	0.57	0.91
Number of observations^a^	71	177	36	131	326	35

^a^Some n’s smaller due to a small amount of missing information. +*p* ≤ 0.10; *p ≤ 0.05; **p ≤ 0.01; ***p ≤ 0.001 for chi-square test between groups

For the satisfaction measures, less difference is observed between the clients who were segmented and those who were not. Where significant differences are found, this generally represents the clients who had missing forms reporting higher satisfaction or shorter wait time as compared to all others.

Table 4 demonstrates that new clients who were segmented report higher quality services (three out of four items and the quality score) than those who were not segmented in both Arms 1 and 2. However, differences by arm in receiving full information about the selected method are not significant in Arm 1. This may be due in part to the small number of observations in this analysis that is limited to new clients. As found for the full sample, little difference is observed among new clients on their satisfaction by whether or not they were segmented.

Table 5 presents four different multivariate models for the quality of care outcomes. For each outcome, the first model is the full sample which compares results by arm to determine if clients visiting CSIs in Arms 1 and 2 report higher quality services than clients visiting CSIs in Arm 3 where segmentation did not take place. Model 2 is the same as Model 1 but focuses only on new clients. Model 3 examines only clients visiting Arm 1 and Arm 2 CSIs to determine if clients who were segmented versus those who were not reported better quality services. Finally, Model 4 is the same as Model 3 but only includes new clients in Arms 1 and 2. All models control for age, education, and parity (not shown, contact first author for full models).

**Table 5:** Multivariate regression results for association between segmentation and quality of care outcomes, Dosso, Niger, 2020

Model	Key variables	Received information about different methods		Asked about method preference		Shown demonstration kit of methods		Interaction with provider		Quality Score
		OR	95% CI	OR	95% CI	OR	95% CI	OR	95% CI	Coef (SE)
Model 1, Full sample	Arm 1	2.93	1.13–7.61*	2.51	1.14–5.51*	4.85	1.63–14.46**	3.59	1.10–11.70*	0.17 (0.07)**
	Arm 2	1.18	0.46–2.97	0.83	0.39–1.74	3.98	1.36–11.64*	2.29	0.71–7.36	0.09 (0.07)
	Arm 3 (ref)	1.00		1.00		1.00		1.00		
	New user	3.97	3.23–4.87***	3.60	2.85–4.56**	3.19	2.57–3.96***	1.39	1.12–1.71**	0.16 (0.01)***
	Continuing user (ref)	1.00		1.00		1.00		1.00		
Model 2, New users on day of interview	Arm 1	3.65	1.17–11.31*	2.09	0.62–7.02	5.97	1.53–23.32**	2.85	0.87–9.32+	0.15 (0.07)*
	Arm 2	1.43	0.49–4.13	0.85	0.28–2.57	6.75	1.79–25.44**	2.76	0.86–8.87+	0.12 (0.07)+
	Arm 3 (ref)	1.00		1.00		1.00		1.00		
	Never used	5.40	3.36–7.96***	10.56	6.16–18.08***	4.75	3.26–6.94***	2.72	1.89–3.92***	0.24 (0.02)***
	Ever use (ref)	1.00		1.00		1.00		1.00		
Model 3, Arms 1 and 2, Full sample	Arm 1	2.08	0.83–5.22	2.59	1.19–5.64*	1.04	0.40–2.72	1.31	0.37–4.62	0.05 (0.06)
	Arm 2 (ref)	1.00		1.00		1.00		1.00		
	New user	3.1	2.30–4.17***	2.2	1.56–3.11***	2.76	2.06–3.69***	1.11	0.83–1.47	0.11 (0.02)***
	Continuing user (ref)	1.00		1.00		1.00		1.00		
	Segmented	5.18	3.75–7.16***	5.59	3.73–8.36***	4.38	3.18–6.04***	2.69	1.97–3.69***	0.25 (0.02)***
	Missing segment	1.6	1.03–2.49*	0.97	0.59–1.59	1.52	0.92–2.51	2.10	1.32–3.32**	0.09 (0.03)***
	Not segmented (ref)	1.00		1.00		1.00		1.00		
Model 4, Arms 1 and 2, new users on day of interview	Arm 1	2.78	0.90–8.63+	3.77	0.97–14.57+	1.00	0.28–3.50	1.02	0.30–3.49	0.03 (0.06)
	Arm 2 (ref)	1.00		1.00		1.00		1.00		
	Never used	5.66	3.17–10-11***	5.15	2.46–10.79***	4.11	2.49–6.79***	3.68	2.45–6.04***	0.22 (0.02)***
	Ever use (ref)	1.00		1.00		1.00		1.00		
	Segmented	4.05	2.35–6.98***	6.65	3.12–14-14***	3.13	1.86–5.26***	1.59	0.95–2.63+	0.17 (0.03)***
	Missing segment	0.96	0.39–2.33	0.27	0.09–0.77*	0.72	0.28–1.85	1.17	0.49–2.83	−0.01 (0.04)
	Not segmented (ref)	1.00		1.00		1.00		1.00		

Note, all models control for age, education, and parity; +*p* ≤ 0.10; *p ≤ 0.05; **p ≤ 0.01; ***p ≤ 0.001. Model 1 n’s: Information: *n* = 2638; Preference: *n* = 2626; Mallette: *n* = 2661; Interaction: *n* = 2623; Score: *n* = 2674. Model 2 n’s: Information: *n* = 1226; Preference: *n* = 1221; Mallette: *n* = 1239; Interaction: *n* = 1236; Score: *n* = 1250. Model 3 n’s: Information: *n* = 1526; Preference: *n* = 1509; Mallette: *n* = 1535; Interaction: *n* = 1512; Score: *n* = 1547. Model 4 n’s: Information: *n* = 742; Preference: *n* = 736; Mallette: *n* = 745; Interaction: *n* = 745; Score: *n* = 755

In Model 1 of Table 5, we see that clients in Arm 1 have higher odds of reporting better quality services (all outcomes) than clients in Arm 3. Further, clients in Arm 2 also have higher odds of seeing the demonstration kit of methods than clients in Arm 3. Clients who were new users on the day of the visit report higher quality services compared to returning users as indicated across all of the outcomes. In the analysis of only new users (Model 2), similar results are found such that new clients in Arm 1 are more likely to have received information about different methods (OR: 3.65; 95% CI: 1.17–11.31), seen the demonstration kit of methods (OR: 5.97; 95% CI: 1.53–23.32), and had better provider interaction (OR: 2.85; 95% CI: 0.87–9.32; *p* < 0.10) than clients in Arm 3. New clients in Arm 2 have higher odds of seeing the demonstration kit of methods (OR: 6.75; 95% CI: 1.79–25.44) and had better provider interactions (OR: 2.76; 95% CI: 0.86–8.87; *p* < 0.10) than new clients in Arm 3. Among new clients on the day of the visit (Model 2), those who had never used a method reported higher quality services (all outcomes) than those who had ever used a method.

The assessments of the role of segmentation for clients in Arms 1 and 2 are shown in Models 3 and 4 and demonstrate that generally, when clients were segmented (either among all clients in Model 3 or among new clients in Model 4), they have higher odds of receiving better quality services than clients who were not segmented. For example, in the full sample from Arms 1 and 2 (Model 3), those clients who were segmented have higher odds of reporting that they were asked about their preference for a method (OR: 5.59, 95% CI: 3.73–8.36) than those clients who were not segmented. Likewise, on the overall quality score, those clients who were segmented report a higher quality score than those clients who were not segmented (β = 0.25; SE = 0.02; *p*-value< 0.001). In addition, among new clients (Model 4), those who were segmented have higher odds of reporting that they were given information about different methods than new clients who were not segmented (OR: 4.05, 95% CI: 2.35–6.98). New clients who were segmented also had a higher overall quality score than new clients who were not segmented. Similar results are found for the other quality measures. Among the control variables, few consistent patterns are found across the four models; however, more educated women have higher odds of being asked their preference than the women with no education.

Table 6 presents the same four models for the satisfaction outcomes of wait time, treatment by the provider, overall satisfaction with the visit, and the satisfaction score. Fewer significant differences are observed in these models. In Model 1, new users have lower odds of reporting satisfaction in terms of wait time (OR: 0.68; 95% CI: 0.54–0.85) but higher odds of reporting being treated very well by the provider (OR: 1.32: 95% CI: 1.07–1.62) than continuing users; similar results are found in Model 3 for the sample from Arms 1 and 2. In Models 3 and 4 that focus on the Arm 1 and 2 samples, no differences are found in satisfaction outcomes between clients who were segmented and those who were not segmented. As shown in the descriptive results, across all models, clients in Arm 2 have a higher overall satisfaction score than clients in Arm 1 and Arm 3. No consistent pattern is found with the control variables in the satisfaction models.

**Table 6:** Multivariate regression results for association between segmentation and satisfaction with service outcomes, Dosso, Niger, 2020

Model	Key variables	Waiting time: no time or reasonable vs. too long		Treated very well by provider		Very satisfied with visit today		Satisfaction Score
		OR	95% CI	OR	95% CI	OR	95% CI	Coef (SE)
Model 1, Full sample	Arm 1	1.77	0.69–4.57	1.17	0.30–4.51	1.19	0.38–3.71	0.03 (0.06)
	Arm 2	1.69	0.68–4.21	2.91	0.77–11.00	2.55	0.83–7.83	0.13 (0.06)*
	Arm 3 (ref)	1.00		1.00		1.00		
	New user	0.68	0.54–0.85**	1.32	1.07–1.62**	1.19	0.96–1.46	0.01 (0.01)
	Continuing user (ref)	1.00		1.00		1.00		
Model 2, New users on day of interview	Arm 1	1.69	0.66–4.34	1.17	0.29–4.66	1.45	0.41–5.19	0.04 (0.08)
	Arm 2	1.46	0.60–3.54	3.30	0.85–12.75+	3.28	0.94–11.39+	0.13 (0.08)+
	Arm 3 (ref)	1.00		1.00		1.00		
	Never used	0.93	0.65–1.34	0.97	0.68–1.39	1.33	0.92–1.94	0.01 (0.02)
	Ever use (ref)	1.00		1.00		1.00		
Model 3, Arms 1 and 2, Full sample	Arm 1	1.04	0.38–2.81	0.42	0.10–1.72	0.50	0.15–1.68	−0.09 (0.05)+
	Arm 2 (ref)	1.00		1.00		1.00		
	New user	0.63	0.45–0.90*	1.32	0.99–1.75+	1.33	0.99–1.78+	0.01 (0.02)
	Continuing user (ref)	1.00		1.00		1.00		
	Segmented	1.03	0.69–1.52	1.11	0.81–1.52	1.12	0.81–1.56	0.01 (0.02)
	Missing segment	1.38	0.80–2.37	0.74	0.45–1.22	0.68	0.40–1.16	−0.01 (0.03)
	Not segmented (ref)	1.00		1.00		1.00		
Model 4, Arms 1 and 2, new users on day of interview	Arm 1	1.13	0.42–3.61	0.37	0.10–1.37	0.46	0.12–1.75	−0.11 (0.04)**
	Arm 2 (ref)	1.00		1.00		1.00		
	Never used	0.98	0.56–1.73	1.09	0.68–1.72	1.83	1.11–3.00*	0.03 (0.03)
	Ever use (ref)	1.00		1.00		1.00		
	Segmented	0.71	0.39–1.30	0.89	0.55–1.43	0.89	0.52–1.50	−0.04 (0.03)
	Missing segment	0.95	0.36–2.51	0.83	0.34–1.97	0.67	0.26–1.68	0.01 (0.05)
	Not segmented (ref)	1.00		1.00		1.00		

Note, all models control for age, education, and parity; +*p* ≤ 0.10; *p ≤ 0.05; **p ≤ 0.01; ***p ≤ 0.001. Model 1 n’s: Wait time: *n* = 2670; Treatment: *n* = 2670; Satisfaction: *n* = 2667; Score: *n* = 2670. Model 2 n’s: Wait time: *n* = 1245; Treatment: *n* = 1245; Satisfaction: *n* = 1243; Score: *n* = 1245. Model 3 n’s: Wait time: *n* = 1543; Treatment: *n* = 1543; Satisfaction: *n* = 1540; Score: *n* = 1543. Model 4 n’s: Wait time: *n* = 750; Treatment: *n* = 750; Satisfaction: *n* = 748; Score: *n* = 750

## Discussion

This study was implemented in Niger where fertility levels remain high and family planning use is low. Identifying approaches to address unmet FP needs in this context is a priority of the Niger government and local implementing partners, including Pathfinder International. Programs in these contexts require multifaceted strategies that include demand creation – i.e., promoting the importance of FP for birth spacing, as well as addressing the quality of services provided (i.e., supply-side programming). Quality improvement programming takes many forms, but one approach focuses on the provision of targeted counseling to clients through strategies like the Balanced Counseling Strategy [16, 19]. This study is the first study in Niger to examine whether segmenting and counseling clients with a targeted approach leads to better quality services and more satisfied clients.

Our study had three arms, one where the segmentation strategy had been implemented since 2017 as part of the Reaching Married Adolescents (RMA) project, one where segmentation had recently been launched, and one without segmentation. Because the RMA project was a community-based demand creation project, the study team determined it was important to have a non-RMA arm with segmentation, in case people in the RMA sites have higher expectations for FP services that would bias the results on quality and satisfaction. Our results demonstrate that the quality of services in Arm 1 was higher than in Arm 2 and Arm 3, and when differences were observed, Arm 2 was also better than Arm 3. That said, clients in Arm 2 were more satisfied than clients in Arm 1; this may reflect the recent training on segmentation leading to improved interactions between providers and clients.

Descriptive results from this study demonstrate continued gaps in service provision in the study sites. While more than three-quarters of women were asked their preference for a method, less than half were provided with complete information on the method that they received. Notably, this difference in receiving full information may reflect that new clients are more likely to receive full information than returning clients who have less need (or demand) for information; however, even among new clients, information provided was less than ideal. Further, while we find no significant descriptive difference in satisfaction across the arms, less than half of clients report that they were “very satisfied” with services and less than half report that they were treated “very well” by the provider. This is indicative of continued improvements needed in the study CSIs.

Our multivariate results demonstrate that clients in facilities where segmentation is being used (Arms 1 and 2) are generally receiving higher quality services. Notably, those clients in Arm 1 where segmentation had been implemented for a longer period of time are the most likely to report receiving quality services whereas for those in Arm 2 where segmentation was a new approach, a smaller number of quality indicators were significant (compared to the comparison arm in Models 1 and 2).

We show that compared to clients who were returning to the facility on the day of service for a reinjection or refill of pills, those who were non-users seeking to adopt a method report receiving better quality services. This suggests that those clients who most need information on a full range of methods are obtaining this information in the Niger study facilities, no matter which study arm they are in. Further, in the sample of new users on the day of service, those who had never used a method in the past are also receiving higher quality information and services than those who have prior experience with a method, no matter the study arm.

Importantly, among clients in the intervention arms where segmentation is being implemented, we found that those clients who were segmented report receiving higher quality services compared to those who were not segmented. This is found in the full sample as well as in the reduced sample of new clients in Arms 1 and 2. Given that segmentation is meant to be targeted to new clients, this last result is important and indicates that if all new clients had been segmented, service quality may have been higher. These results suggest that segmentation is associated with greater counseling and targeted services for clients. In qualitative results from in-depth interviews with providers from facilities where segmentation is underway, providers felt that the segmentation tool and counseling cards lead to them spending more time with clients and the clients feeling more informed and comfortable with their method at the end of their FP visit [28].

In our analyses of satisfaction of services that is captured through the wait time, how the provider treated the clients, and overall satisfaction with the visit, fewer differences between groups are observed. As mentioned above, clients in Arm 2 report being more satisfied (satisfaction score) than clients in Arm 1 and Arm 3. In models with the full sample, new clients (versus continuing users) had longer wait times; however, they were also more likely to report being treated very well or were very satisfied. No difference is found on these satisfaction measures when the sample was limited to only new clients. Finally, no difference is found between segmented and non-segmented clients on any of the satisfaction outcomes. These null results may reflect that there are truly no differences between these groups on satisfaction or alternatively that these satisfaction measures show little variation across groups and thus might not be capturing these outcomes in a manner that is salient to the clients.

The results of this study are similar to earlier studies that examine targeted counseling interventions. In particular, the segmentation strategy that is being implemented in the Niger study facilities is a targeted counseling approach that is similar to the Balanced Counseling Strategy (BCS) that was developed, implemented and tested in Central and South America by colleagues at the Population Council [16]. Earlier studies of BCS demonstrated that when the BCS tools were used, clients had greater knowledge of FP, and service quality was better in terms of interpersonal relations, choice of methods, provision of information on side effects, and information on method selected. Further, the intensity of the provider training on the BCS tools was also related to FP service quality outcomes [16, 17]. This might explain the differences found here between Arms 1 and 2 whereby in Arm 1, the intervention had been ongoing for a longer period of time, so providers had received more supervision visits and support compared to Arm 2 where providers were more recently trained and had only been implementing the segmentation strategy for a short time prior to the study launch. This is consistent with prior studies that have demonstrated that supportive supervision can improve the quality of health care services by increasing provider motivation and permitting providers to better communicate challenges with their supervisor [29, 30]. Thus, in this case, it may be that a combination of the segmentation strategy and supportive supervision leads to better quality services than the standard of care (Arm 3) and to the recently trained sites (Arm 2). An alternative explanation for the better quality of services in Arm 1 and Arm 2 compared to the comparison arm (Arm 3) may relate to the implementation of any training of providers, not specifically the segmentation training. When providers receive training, this often affects their behaviors at least in the short-term. In this study, results from Arm 1 suggest that improved service quality behaviors continued into the longer-term, potentially related to follow-up training and continual program supervision in the Pathfinder RMA sites. Besides the operations research studies in Peru and Guatemala led by Population Council, other studies testing targeted counseling approaches typically did not include a comparison group [19–22]; this is a strength of this study that includes sites where segmentation was not implemented.

This study is not without limitations. A key limitation of this study is that we are not able to determine if identified improvements in the quality of care outcomes are specific to the use of the segmentation approach or if they reflect that the providers in the intervention arms were recently trained (and received recent supervision visits to refresh their engagement). This is a limitation of this study design that did not include a refresher family planning training in Arm 3. Examining provider skills and use of the segmentation tool and counseling cards at a later period (i.e., one year post segmentation training) might provide insights into whether this strategy is adopted by providers and considered the new standard of practice or if they go back to their prior approaches once training and routine supervision ends. Second, the measure of whether the client (in Arm 1 or Arm 2) was segmented does not necessarily reflect segmentation on the day of the visit, particularly in Arm 1 that had been implementing segmentation for a longer duration. Thus, for those clients who were returning on the day of the visit and were segmented at an earlier visit, the earlier segmentation experience likely does not affect their quality of care or satisfaction on the day of the visit. By reducing the sample to new clients, our results attempt to reduce this bias by focusing on those clients in Arm 1 and 2 that were meant to be segmented on the day of the visit. Relatedly, for those with missing segmentation sheets, we are unable to say if they were or were not segmented on the day of the visit or at an earlier clinic visit; this was a consequence of having to withdraw from fieldwork one week early before we were able to check all of the clinic records. Third, as noted above, the null results around satisfaction with services may reflect truly null results or they may reflect challenges with measuring this subjective concept. Alternatively, the satisfaction results may reflect specific client-provider interactions whereby trained (and untrained) providers continue to have biases toward some clients (e.g., unmarried or youth clients) and these are reflected in the outcomes reported at the individual level; we are unable to control for the specific provider that the client met with to adjust for this potential bias. Future studies are needed that attempt to better refine our definition and measurement of client satisfaction to better assess the effect of segmentation (and other quality improvement strategies) on client satisfaction. Fourth, the results on the quality of services received are based on clients’ recollection of their visit. If the provider discussed a topic but it was not of interest or importance to the client, she may not have reported being exposed. Alternatively, clients may have also over-reported provider engagement during their clinic visit if they thought this was the appropriate response. With the data available, it is not possible to know the effect of this self-reporting on the quality of care outcomes. Fifth, as mentioned above, we were not able to identify which provider each client saw and therefore, we were not able to adjust for provider-specific differences in client experience. We adjust for facility-level clustering in the models to at least control for the fact that clients come from a sample of 45 facilities and are not completely independent of one another. Finally, while this study captures perspectives of FP clients who were visiting to get counseling or services, these women who overwhelmingly adopted a method (or continued a method) are not representative of the general population of Niger where only 18% of married women are using a method. If perceived quality of services is a barrier to use, the reports of actual clients will not represent the perspectives of the general population.

## Conclusions

This study is timely since the Government of Niger is considering scaling-up the segmentation approach nationally. Our results suggest that training providers on the segmentation tool and the counseling cards and having follow-up supervision visits can lead to improved quality of services. That said, our results suggest that the Government of Niger will want to consider strategies for the long term to obtain better outcomes. For example, clients in Arm 1 where there was more intensive supervision and follow-up and the segmentation strategy had been underway since 2017 received higher quality services than clients in Arm 2 where providers had only recently been trained and thus had less experience with the segmentation approach. To support scale-up, it is important to identify ways to make segmentation part of the standard of practice and part of routine supervision otherwise there is a risk that once the segmentation forms (i.e., the 12-question sheet) are stocked out or the counseling cards are lost, that service provision is no longer targeted and specific to client needs. Improving quality of services is no easy task but the Government of Niger should make it a priority through this segmentation approach or another approach that strengthens provider skills and supports continued targeted services to meet all client needs. These types of quality improvement programs will help the government attain its FP2020/FP2030 and Sustainable Development Goals to increase modern contraceptive use.

## Data Availability

Data are available at: https://dataverse.unc.edu/dataset.xhtml?persistentId=doi:10.15139/S3/Y4E0YX.

## Abbreviations

BCS: Balanced Counseling Strategy
BCS +: Balanced Counseling Strategy Plus.
BC: Balanced counseling
CHW: Community health worker
CI: Confidence interval
CNERS: Comité National d’Ethique pour la Recherche en Santé
COVID-19: Novel coronavirus
CSI: Centre de Santé Intégré
FP: Family planning
LARC: Long-acting reversible contraceptives
OR: Odds ratio
RMA: Reaching Married Adolescents
TFR: Total fertility rate
USAID: United States Agency for International Development

## Footnotes

1 See http://www.cambercollective.com/fpniger for these resources.
